# Causal association of metabolic biomarkers and the risk of esophageal cancer: A 2-sample Mendelian randomization study

**DOI:** 10.1097/MD.0000000000043295

**Published:** 2025-08-01

**Authors:** Liu Ziyang, Jinzhou Guo, Wenxuan Du

**Affiliations:** a Academy of Zhongjing, Henan University of Chinese Medicine, Zhengzhou, China; b Laboratory of TCM Syndrome and Prescription Signaling, Academy of Zhongjing, Zhengzhou, Henan, China.

**Keywords:** blood metabolite, causal inference, esophageal cancer, MR analysis

## Abstract

Esophageal cancer (EC) is a major malignancy with poor prognosis and a 5-year survival rate below 30%. Metabolites are key biomarkers for cancer diagnosis, prognosis, and treatment. Their changes and metabolic reprogramming are crucial in EC progression. However, traditional studies struggled to determine causality. A two-sample Mendelian randomization (MR) analysis was performed to determine the causal association between blood metabolites and EC. Using publicly available genetic data, causal associations between 1400 blood metabolites and EC risk were explored. Multiple MR estimation techniques were incorporated, including inverse variance weighting (IVW), MR Egger regression, weighted median, weighted mode, and simple mode. Additionally, sensitivity analyses were conducted to assess the reliability of the results. Using strict inclusion criteria and sensitivity analyses, 9 blood metabolites were identified as significantly associated with EC risk. However, reverse MR analysis indicated potential reverse causality for tyrosine levels, narrowing the focus to 8 metabolites. The identified metabolites and their associations with EC risk were: maltotriose levels (IVW odds ratio [OR] = 1.2396, 95% CI = 1.0658–1.4418, *P* = .0053); 3-hydroxy-2-ethylpropionate levels (IVW OR = 0.7760, 95% CI = 0.6631–0.9081, *P* = .0016); 5-dodecenoate (12:1n7) levels (IVW OR = 1.4176, 95% CI = 1.0908–1.8421, *P* = .0090); 3-methyladipate levels (IVW OR = 0.7469, 95% CI = 0.6125–0.9108, *P* = .0039); 8-methoxykynurenate levels (IVW OR = 1.2246, 95% CI = 1.0654–1.4076, *P* = .0043); spermidine to 5-methylthioadenosine ratio (IVW OR = 1.2558, 95% CI = 1.0610–1.4864, *P* = .0081); adenosine 5’-diphosphate to sulfate ratio (IVW OR = 1.1923, 95% CI = 1.0435–1.3622, *P* = .0097); glucose-to-mannose ratio (IVW OR = 0.8153, 95% CI = 0.7091–0.9375, *P* = .0042). Our study has demonstrated the close connection between blood metabolites and EC by genetic means, thus providing guidance for future clinical research.

## 1. Introduction

Esophageal cancer (EC) is a significant public health concern due to its high incidence and poor prognosis.^[1]^ As one of the most common malignancies in the digestive system, EC ranks 8th in terms of incidence and 6th in mortality among all malignant tumors globally.^[2]^ The disease is characterized by the dysplasia of esophageal epithelial or squamous epithelium, due to the expansion and muscular nature of the esophagus, early-stage EC patients may not recognize symptoms of obstruction or stenosis.^[3]^ Symptoms only appear when the tumor has locally progressed or even metastasized.^[4]^ In the United States and Europe, the majority of EC patients are diagnosed at a locally advanced or metastatic stage, making them unsuitable for radical treatment.^[5]^ Consequently, the overall 5-year survival rate for EC is <30%,^[6]^ and for advanced cases, it drops to a mere 5%.^[7]^ The high mortality and low survival rates highlight the importance of early diagnostic indicators and new treatment approaches.

Metabolites, the stable end products of various metabolic pathways, have emerged as crucial biomarkers for cancer diagnosis, prognosis, and treatment evaluation. Tumor cells exhibit distinct metabolic profiles compared to normal cells, with metabolic disorders considered hallmarks of cancer.^[8]^ For instance, a primary tumor model experiment identified 52 metabolites with differential expression compared to normal cells.^[9]^ In EC, significant alterations in carbohydrate, amino acid, and lipid metabolism have been documented.^[10]^ These findings highlight that metabolic reprogramming is integral to cancer progression, underscoring the potential of metabolites as diagnostic markers and therapeutic targets.^[11]^ Although observational studies have provided insights into the associations between metabolites and cancer, they are inherently limited in establishing causality. For example, significant differences have been observed in the concentrations of amino acids and lipids between EC patients and healthy controls.^[9]^ Additionally, tumor cells upregulate the glycolytic pathway during their constant adaptation to the dynamic metabolic microenvironment, leading to rapid growth.^[12]^ These studies can identify correlations; however, they cannot definitively determine whether metabolic changes cause cancer or are a consequence of it. This limitation arises primarily due to confounding factors and reverse causation, which are challenging to control in traditional epidemiological research.

Mendelian randomization (MR) provides a robust method to infer causal relationships by utilizing genetic variants, specifically single nucleotide polymorphisms (SNPs), as instrumental variables (IVs).^[13,14]^ MR leverages the random assortment of genes from parents to offspring, mimicking the randomization process in clinical trials. This approach effectively mitigates confounding factors and reverse causation, thus providing more reliable causal inferences about the effects of modifiable risk factors on disease outcomes.^[15]^ Recent advancements in genomic technologies and the availability of large-scale genome-wide association studies (GWAS) databases have further enhanced the application of MR in cancer research. This study employs a bidirectional two-sample MR approach to investigate the causal relationship between specific metabolites and the risk of EC. By leveraging large-scale GWAS data, we aim to identify metabolites that may serve as early diagnostic biomarkers or therapeutic targets. This research not only deepens our understanding of the metabolic underpinnings of EC but also opens new avenues for developing alternative therapies and improving patient outcomes.

Furthermore, this study reflects a broader perspective of inverse inference, wherein disease risk is inferred from molecular biomarker data. This concept aligns with the mathematical framework of inverse problems, which has been widely applied in various scientific disciplines. For example, inverse problems have been studied in the context of predator–prey models,^[16]^ spectral analysis with incomplete data,^[17]^ and multi-population aggregation systems.^[18]^ Incorporating such perspectives may inspire innovative methodological approaches for biomarker-driven disease modeling and deepen interdisciplinary collaboration between biomedical research and mathematical theory.

## 2. Materials and methods

### 2.1. Study design

**Figure 1.**
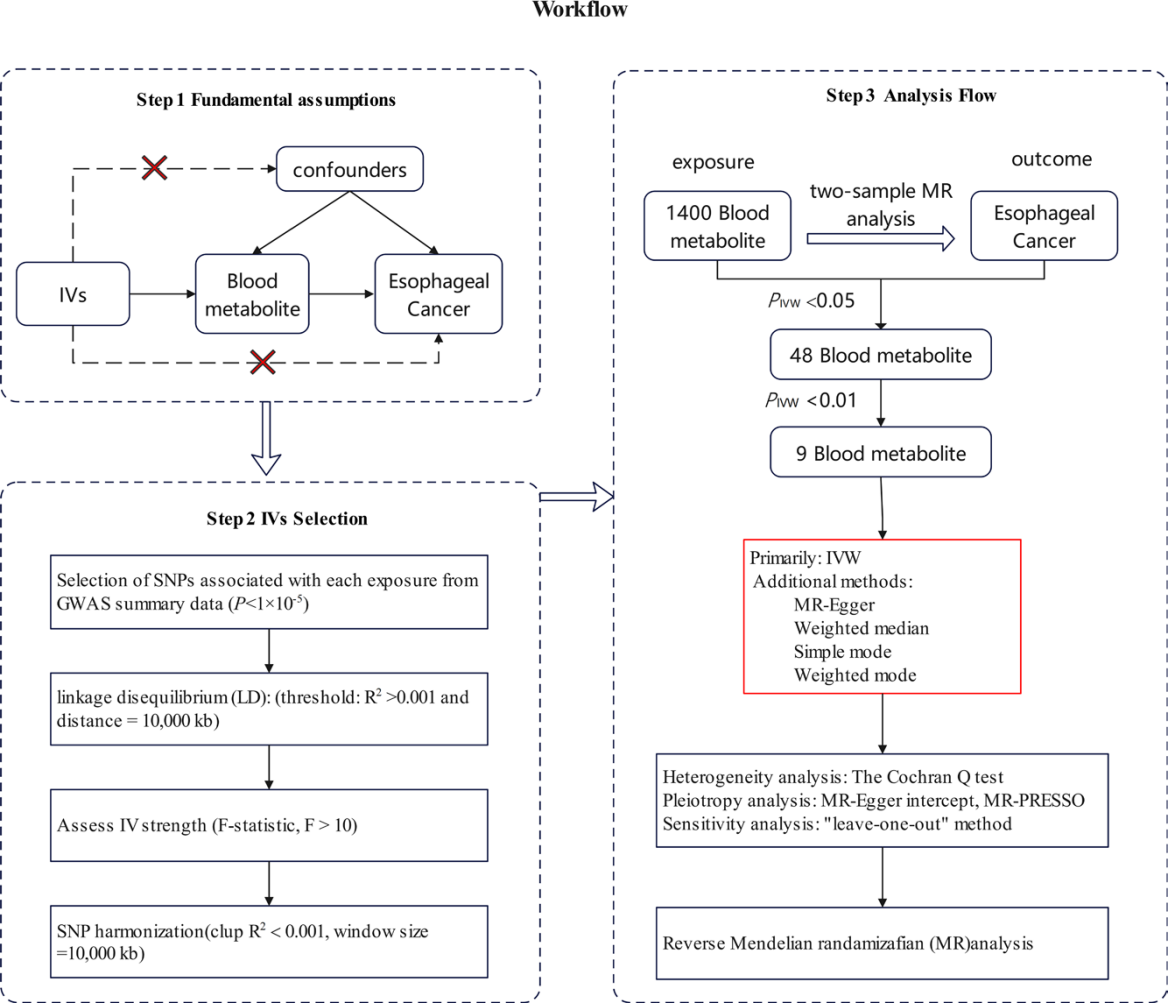
The steps of Mendelian randomization (MR) analysis. IVW = inverse variance weighting, LD = linkage disequilibrium.

The present study utilized a bidirectional two-sample MR approach to explore the causal relationships between 1400 blood metabolites and EC. To ensure robust causal inference, the IVs were required to meet 3 fundamental assumptions^[14]^: (1) each IV must be directly associated with the exposure factors. (2) Each IV should not be correlated with any potential confounders between the exposure and the outcome. (3) Each IV should influence the outcome solely through the exposure and not through any other pathway. All MR analyses were performed using publicly available summary statistics, thereby eliminating the need for additional ethical approval or informed consent. The analytical principles and procedures are depicted in Figure 1.

### 2.1. Data source

The GWAS summary statistics for EC were accessed from the GWAS Catalog under accession number GCST90018841.^[19]^ This GWAS included 160,589 individuals of predominantly European ancestry, with 1388 cases and 159,201 controls. Similarly, the summary statistics for 1400 blood metabolites were obtained from the GWAS Catalog under accession numbers GCST90199621 to GCST90201020^[20]^ (see Table S1, Supplemental Digital Content, https://links.lww.com/MD/P385).

### 2.2. IV selection

To enhance the reliability of IVs and ensure the stability of study data and accuracy of results, IVs were selected based on the following criteria:

Firstly, relevance to exposure: SNPs weakly correlated or uncorrelated with the exposure (*P* > 1e-5) were excluded; secondly, linkage disequilibrium (LD): to eliminate LD among IVs and meet the requirements of MR analysis, IVs had to satisfy the conditions of *R*² < 0.001 and LD = 10,000 kb^[21]^; thirdly, instrument strength: the strength of genetic variation as IVs was assessed using the F-statistic, with a screening criterion of F > 10.^[22]^ The F-statistic was calculated using the formula:


$$\begin{array}{l} F=\frac{\left(N-K-1\right)\cdot \;\;\;R^{2}}{K\cdot \;\;\;\left(1-R^{2}\right)} \end{array}$$

where N is the sample size in the exposure database, K is the number of IVs, and *R*^2^ is the proportion of variance explained by SNPs in the exposure database. The formula for calculating *R*^2^ is:


$$\begin{array}{l} R^{2}=\frac{\beta^{2}\cdot EAF\cdot \left(1-EAF\right)}{SE^{2}\cdot N} \end{array}$$

where β is the allele effect value, EAF is the effect allele frequency, SE is the standard error, and N is the sample size; finally, data harmonization: when combining the exposure and outcome datasets, incompatible alleles and palindromic SNPs were eliminated to ensure data consistency.^[23]^

### 2.3. MR analysis

The R software package “TwoSampleMR” was used to perform a two-sample MR analysis to explore the causal relationship between 1400 blood metabolites and EC. The primary method employed was IVW.^[24]^ To ensure the reliability and consistency of the results, 4 additional methods were applied: simple mode, weighted median, weighted mode, and MR Egger.^[25]^ Reverse MR validations were conducted to enhance the robustness and practical significance of the findings.

### 2.4. Sensitivity analysis

The positive findings from the MR analysis were thoroughly validated through several tests: heterogeneity testing, horizontal pleiotropy testing, and leave-one-out sensitivity analysis, aimed at increasing confidence in the results. Heterogeneity among individual estimates of genetic variation was assessed using the Cochran Q test.^[26]^ No significant heterogeneity was found (*P* > .05). Horizontal pleiotropy was evaluated using the MR Egger intercept. The MR Egger method included an intercept term in the regression analysis, allowing a comparison with the IVW method to detect horizontal pleiotropy among IVs.^[27]^ If *P* > .05, indicating the absence of horizontal pleiotropy, the IVW method was used for the MR analysis outcome. The MR-Pleiotropy RESidual Sum and Outlier (MR-PRESSO) method was employed to detect and correct for horizontal pleiotropy and to identify potential outliers.^[28]^ It consists of 3 components: the MR-PRESSO Global Test, which assessed overall horizontal pleiotropy, where a significant *P*-value (*P* < .05) indicated the presence of pleiotropy; the MR-PRESSO Outlier Test, which identified and removed outliers that could bias the causal estimate; and the MR-PRESSO Distortion Test, which evaluated the difference in causal estimates before and after outlier removal to determine if the correction significantly altered the results. A non-significant *P*-value (*P* > .05) suggested that the causal estimate remained stable after outlier correction. The MR-PRESSO analysis was performed using the R package “MRPRESSO.” Sensitivity analysis was conducted using a leave-one-out approach to determine the influence of individual IVs on the overall MR analysis results, providing robust evidence for the stability of the findings.

## 3. Results

To ensure the strength of the instrumental variables, we first calculated the F-statistics for each SNP and included only those with F > 10 to avoid weak instrument bias. As a result, we selected 34,843 SNPs associated with 1400 blood metabolites and 4 SNPs associated with EC as instrumental variables. Detailed characteristics of the SNPs, including *R*² and F-statistics, are provided in Table S2, Supplemental Digital Content, https://links.lww.com/MD/P385.

### 3.1. Exploration of the causal effect of blood metabolites on EC

**Figure 2.**
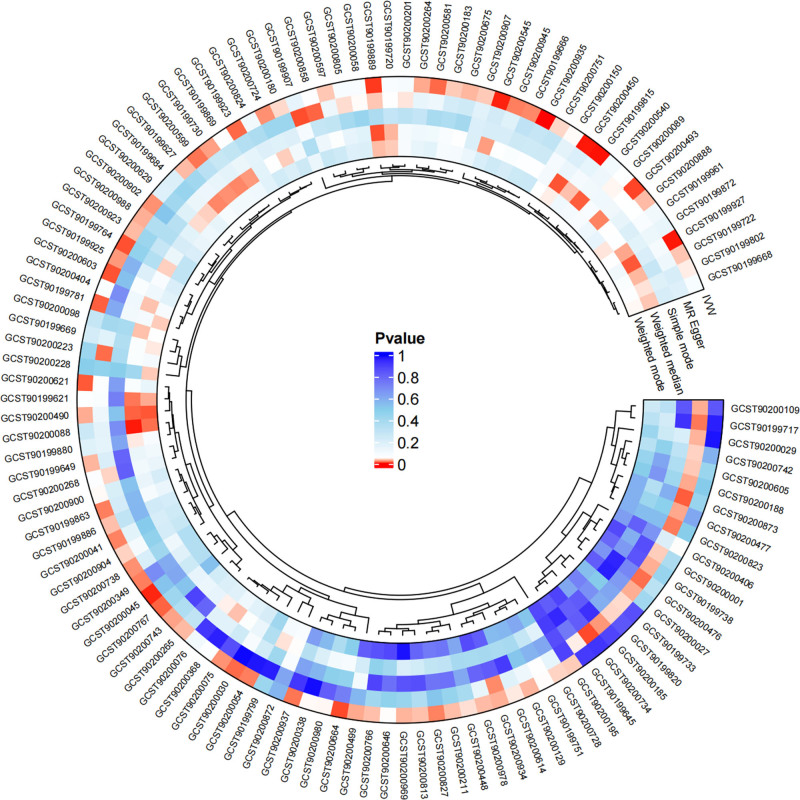
Circle heat map showing significantly causal relationships between 48 blood metabolites and EC. EC = esophageal cancer.

**Figure 3.**
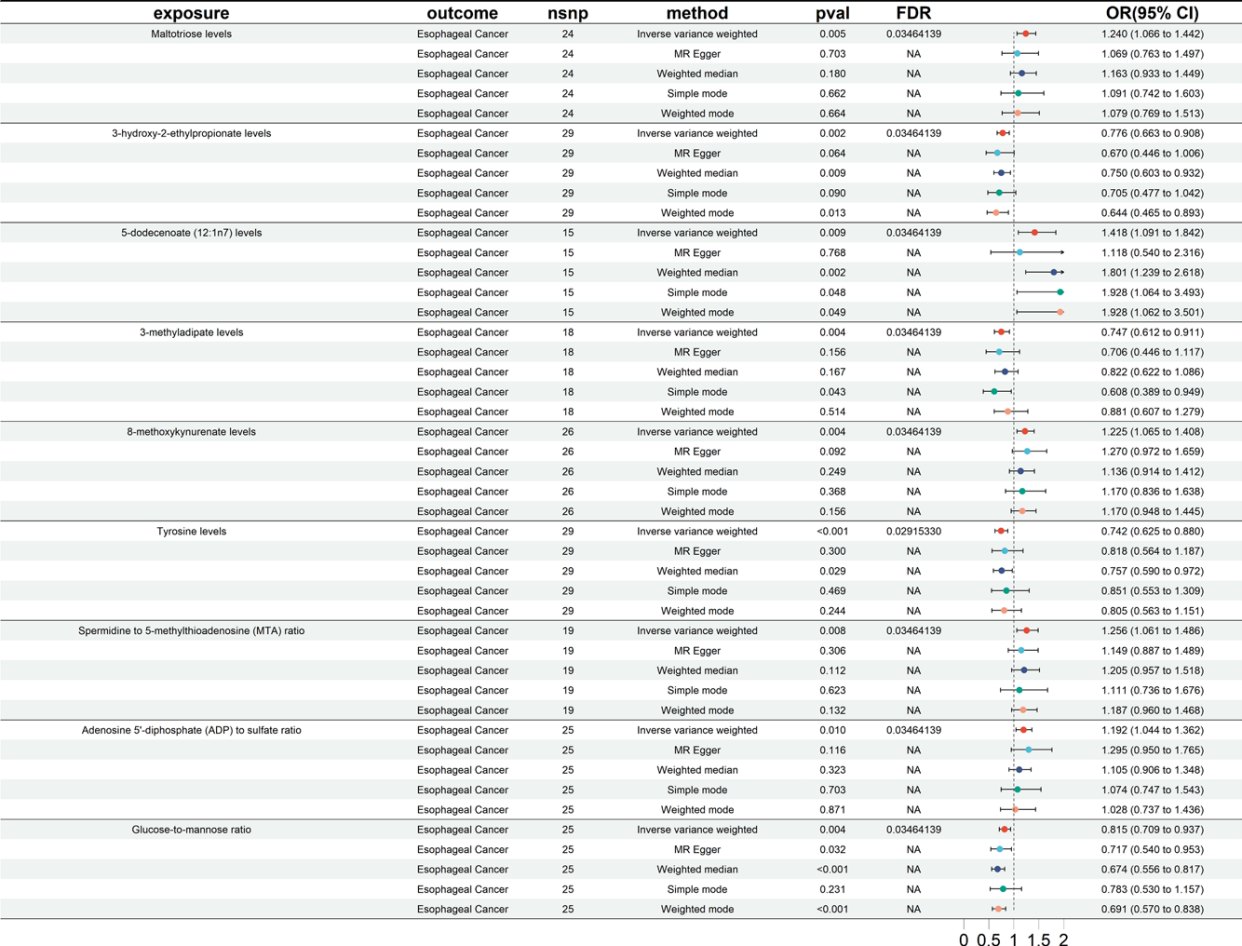
Forest plots showing significantly causal relationships between 9 blood metabolites and EC. CI = confidence interval, EC = esophageal cancer, nSNP = number of single nucleotide polymorphism, OR = odds ratio.

The tests revealed no significant heterogeneity and horizontal pleiotropy among the IVs (*P* > .05) (Table 1). Thus, the outcomes of the IVW analysis were employed as the results of the MR analysis, with a fixed-effects model utilized to present the final findings. The MR analysis results indicate that 48 blood metabolites have a significant causal relationship with EC (*P* < .05) (Fig. 2). To reduce false-positive results, we further screened these metabolites using a more stringent threshold of *P* < .01, and identified 9 blood metabolites with a significant causal association with EC. Specifically including: (1) maltotriose levels (IVW odds ratio [OR] = 1.2396, 95% CI = 1.0658–1.4418, *P* = .0053); (2) 3-hydroxy-2-ethylpropionate levels (IVW OR = 0.7760, 95% CI = 0.6631–0.9081, *P* = .0016); (3) 5-dodecenoate (12:1n7) levels (IVW OR = 1.4176, 95% CI = 1.0908–1.8421, *P* = .0090); (4) 3-methyladipate levels (IVW OR = 0.7469, 95% CI = 0.6125–0.9108, *P* = .0039); (5) 8-methoxykynurenate levels (IVW OR = 1.2246, 95% CI = 1.0654–1.4076, *P* = .0043); (6) tyrosine levels (IVW OR = 0.7417, 95% CI = 0.6252–0.8799, *P* = .0006); (7) spermidine to 5-methylthioadenosine (MTA) ratio (IVW OR = 1.2558, 95% CI = 1.0610–1.4864, *P* = .0081); (8) adenosine 5’-diphosphate (ADP) to sulfate ratio (IVW OR = 1.1923, 95% CI = 1.0435–1.3622, *P* = .0097); (9) glucose-to-mannose ratio (IVW OR = 0.8153, 95% CI = 0.7091–0.9375, *P* = .0042) (Fig. 3). Scatter plots were utilized to illustrate the causal effect estimates (Figure S1, Supplemental Digital Content, https://links.lww.com/MD/P384). Additionally, the robustness of the MR analysis results was assessed by leave-one analysis (Figure S2, Supplemental Digital Content, https://links.lww.com/MD/P384) and funnel plot (Figure S3, Supplemental Digital Content, https://links.lww.com/MD/P384).

**Table 1:** Sensitivity analysis of causal relationships between 9 blood metabolites and EC.

Exposure	Outcome	Method	Cochran Q			Pleiotropy		
			Q	Q_df	Q_pval	Egger intercept	SE	*P* val
Maltotriose levels	EC	MR Egger	19.52	22	0.61	0.0236	0.0245	.34
		IVW	20.45	23	0.61			
		MR-PRESSO						.63
3-Hydroxy-2-ethylpropionate levels	EC	MR Egger	21.85	27	0.74	0.0172	0.0223	.45
		IVW	22.44	28	0.76			
		MR-PRESSO						.75
5-Dodecenoate (12:1n7) levels	EC	MR Egger	13.55	13	0.41	0.0253	0.0368	.50
		IVW	14.04	14	0.45			
		MR-PRESSO						.49
3-Methyladipate levels	EC	MR Egger	6.81	16	0.98	0.0070	0.0262	.79
		IVW	6.88	17	0.99			
		MR-PRESSO						.99
8-Methoxykynurenate levels	EC	MR Egger	28.48	24	0.24	-0.0059	0.0187	.76
		IVW	28.60	25	0.28			
		MR-PRESSO						.34
Tyrosine levels	EC	MR Egger	24.99	27	0.57	-0.0129	0.0222	.57
		IVW	25.33	28	0.61			
		MR-PRESSO						.67
Spermidine to 5-methylthioadenosine (MTA) ratio	EC	MR Egger	21.05	17	0.22	0.0168	0.0189	.39
		IVW	22.03	18	0.23			
		MR-PRESSO						.33
Adenosine 5’-diphosphate (ADP) to sulfate ratio	EC	MR Egger	15.31	23	0.88	-0.0149	0.0258	.57
		IVW	15.65	24	0.90			
		MR-PRESSO						.89
Glucose-to-mannose ratio	EC	MR Egger	25.06	23	0.35	0.0212	0.0209	.32
		IVW	26.18	24	0.34			
		MR-PRESSO						.24

EC = esophageal cancer, IVW = inverse variance weighted, MR-PRESSO = MR-Pleiotropy Residual Sum and Outlier.

### 3.2. Reverse MR analysis

**Figure 4.**
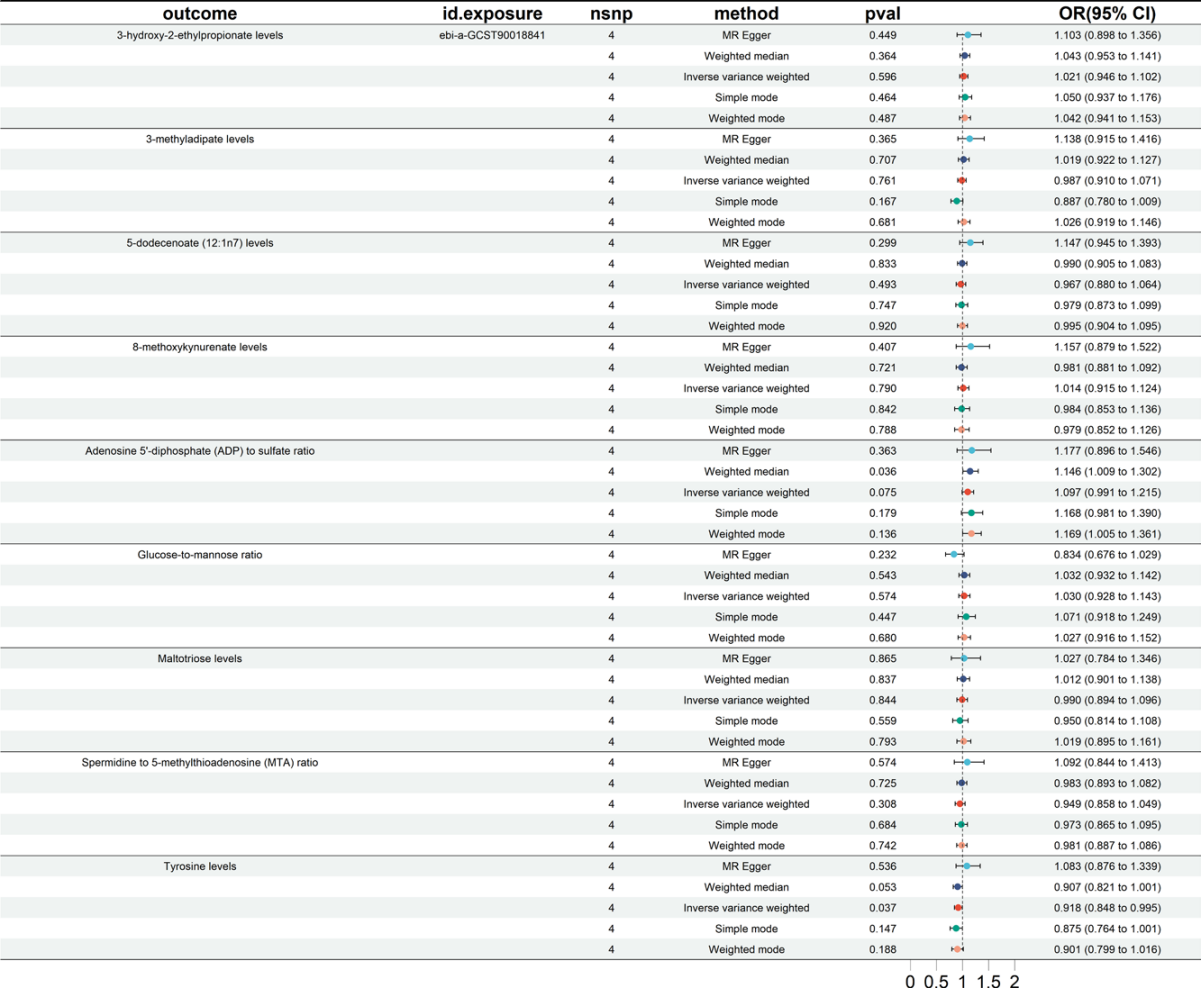
Forest plots showing causal relationships between EC and 9 blood metabolites. CI = confidence interval, EC = esophageal cancer, nSNP = number of single nucleotide polymorphism, OR = odds ratio.

Reverse MR analysis was undertaken to verify the positive findings. In the absence of any abnormalities in heterogeneity and horizontal pleiotropy analyses (Table 2), the MR analysis results indicated a significant causal relationship between EC and 1 blood metabolite. Tyrosine levels (IVW OR = 0.918, 95% CI = 0.848–0.995, *P* = .037) (Fig. 4). Scatter plots were utilized to illustrate the causal effect estimates (Figure S4, Supplemental Digital Content, https://links.lww.com/MD/P384). Additionally, the robustness of the MR analysis results was assessed by leave-one-out analysis (Figure S5, Supplemental Digital Content, https://links.lww.com/MD/P384) and funnel plot (Figure S6, Supplemental Digital Content, https://links.lww.com/MD/P384).

**Table 2:** Sensitivity analysis of causal relationship between EC and 9 blood metabolites.

Outcome	Exposure	Method	Cochran Q			Pleiotropy		
			Q	Q_df	Q_pval	Egger intercept	SE	*P* val
Maltotriose levels	EC	MR Egger	0.45	2	0.80	-0.0123	0.0427	.80
		IVW	0.53	3	0.91			
		MR-PRESSO						.89
3-Hydroxy-2-ethylpropionate levels	EC	MR Egger	1.12	2	0.57	-0.0255	0.0322	.51
		IVW	1.75	3	0.63			
		MR-PRESSO						.66
5-Dodecenoate (12:1n7) levels	EC	MR Egger	1.82	2	0.40	-0.056	0.0302	.20
		IVW	5.26	3	0.15			
		MR-PRESSO						.27
3-Methyladipate levels	EC	MR Egger	0.91	2	0.64	-0.0468	0.0341	.30
		IVW	2.79	3	0.42			
		MR-PRESSO						.47
8-Methoxykynurenate levels	EC	MR Egger	2.96	2	0.23	-0.0435	0.0429	.42
		IVW	4.48	3	0.218			
		MR-PRESSO						.308
Tyrosine levels	EC	MR Egger	0.44	2	0.80	-0.0542	0.0330	.24
		IVW	3.14	3	0.37			
		MR-PRESSO						.44
Spermidine to 5-methylthioadenosine (MTA) ratio	EC	MR Egger	2.73	2	0.26	-0.0460	0.0402	.37
		IVW	4.52	3	0.21			
		MR-PRESSO						.34
Adenosine 5’-diphosphate (ADP) to sulfate ratio	EC	MR Egger	1.74	2	0.42	-0.0232	0.0428	.64
		IVW	2.04	3	0.56			
		MR-PRESSO						.557
Glucose-to-mannose ratio	EC	MR Egger	0.87	2	0.65	0.0695	0.0327	.17
		IVW	5.40	3	0.14			
		MR-PRESSO						.26

EC = esophageal cancer, IVW = inverse variance weighted, MR-PRESSO = MR-Pleiotropy Residual Sum and Outlier.

## 4. Discussion

This study utilized genetic variation data from GWAS to investigate the causal relationships between 1400 blood metabolites and EC. Following stringent inclusion criteria and sensitivity analyses, we identified 9 blood metabolites that exhibit a significant causal relationship with the risk of EC. However, reverse MR indicated a potential reverse causality for tyrosine levels, ultimately narrowing our focus to 8 metabolites of interest. These include maltotriose levels, 3-hydroxy-2-ethylpropionate levels, 5-dodecenoate (12:1n7) levels, 3-methyladipate levels, 8-methoxykynurenate levels, spermidine to MTA ratio, ADP to sulfate ratio, and glucose-to-mannose ratio. The results suggest that these metabolites may play significant roles in the development of EC, offering potential diagnostic and therapeutic targets. Based on the characteristics of these 8 metabolites, we classified them into categories such as carbohydrate metabolism, fatty acid and lipid metabolism, amino acid and derivative metabolism, and polyamines.

Carbohydrate metabolism involves the breakdown, synthesis, storage, and utilization of sugars, which serve as a primary energy source for cells.^[29]^ Central to this is glucose metabolism, which encompasses glycolysis, glycogen synthesis and breakdown, and the pentose phosphate pathway.^[30]^ Abnormal glucose metabolism, known as the Warburg effect, is characterized by preferential aerobic glycolysis even in the presence of oxygen and is often exhibited by tumor cells.^[31]^ This metabolic reprogramming supports cancer cell growth and survival by providing energy and metabolic intermediates.^[32]^ Dysregulated carbohydrate metabolism is a hallmark of many cancers, including EC. These alterations significantly influence tumor growth, invasion, and metastasis.^[33]^ Maltotriose, an oligosaccharide composed of 3 glucose molecules linked by α-1,4 glycosidic bonds,^[34]^ is a breakdown product of starch and glycogen and has played a crucial role in carbohydrate metabolism. Our findings showed that maltotriose levels were positively associated with EC risk (β = 0.2148, *P* = .0053, OR = 1.2396), suggesting that elevated maltotriose levels may increase the risk of EC. This underscored the importance of dysregulated carbohydrate metabolism in the development of EC and highlighted maltotriose levels as a potential biomarker. Additionally, glucose and mannose, monosaccharides involved in sugar metabolism, have played vital roles in carbohydrate metabolism. Altered glucose–mannose ratios impacted glycolytic effects in tumor cells, thereby reducing their energy supply and inhibiting growth and proliferation.^[35]^ Our analysis revealed a negative correlation between the glucose-to-mannose ratio and EC risk (β = -0.2042, *P* = .0042, OR = 0.8153), indicating that higher glucose-to-mannose ratio might reduce EC risk. This finding further illustrated the significance of metabolic reprogramming in cancer progression and suggested that maintaining a balanced glucose–mannose ratio could be a strategy for reducing EC risk.

Fatty acid and lipid metabolism encompasses the synthesis, breakdown, and transport of fatty acids, which are essential for cell membrane structure, energy storage, and signal transduction.^[36]^ Fatty acids undergo β-oxidation to produce energy, while phospholipids and cholesterol are crucial membrane components.^[37]^ Abnormal fatty acid metabolism is a hallmark of many cancers, with tumor cells often displaying altered fatty acid synthesis and oxidation pathways to support proliferation and survival.^[38]^ Key enzymes in fatty acid synthesis, such as fatty acid synthase, are overexpressed in various cancers and correlate with poor prognosis.^[38,39]^ Our study found that serum levels of 5-dodecenoic acid (12:1n7) were positively associated with the risk of EC (β = 0.3489, *P* = .0090, OR = 1.4176). This suggests that this lipid metabolite may play a potential role in the development and progression of EC. Previous studies have shown that lipid metabolic reprogramming in EC often involves dual activation of fatty acid synthesis pathways (such as ACC and fatty acid synthase) and fatty acid degradation pathways (such as CPT1A and ACSL1).^[40,41]^ Lipidomic analyses have further demonstrated significant dysregulation of glycerophospholipid metabolism in EC.^[42]^ In addition, key enzymes involved in fatty acid metabolism, including CPT1A and ACSL1, are abnormally expressed in EC, which may accelerate the breakdown of fatty acids, especially monounsaturated fatty acids (MUFAs), to meet the high energy and membrane synthesis demands of tumor cells.^[40,43]^ 5-Dodecenoic acid (12:1n7) is an omega-7 monounsaturated fatty acid.^[44]^ Its chain length (C12:1) and double bond position may influence its metabolic fate in the body. A previous study^[45]^ indicated that medium-chain fatty acids (such as C12) are more readily oxidized than long-chain fatty acids, and are preferentially used for energy production. Although direct experimental evidence for the role of 5-dodecenoic acid in EC is limited, its characteristics as a C12 MUFA suggest that it may be more easily utilized by cancer cells and enter β-oxidation pathways to support energy production and membrane biosynthesis. CPT1A is a rate-limiting enzyme in fatty acid β-oxidation.^[46]^ It facilitates the transport of long-chain fatty acids into mitochondria for oxidation. Under metabolic stress, such as glucose deprivation or hypoxia, CPT1A helps tumor cells maintain energy supply.^[47]^ In esophageal squamous cell carcinoma, high expression of CPT1A is closely related to tumor metastasis and poor prognosis. CPT1A promotes fatty acid oxidation (FAO), increases intracellular ATP levels, and reduces reactive oxygen species accumulation, thereby enhancing cell survival.^[48]^ Inhibition of CPT1A can also improve treatment sensitivity and reverse drug resistance in tumor cells.^[49]^ ACSL1 is another key enzyme in fatty acid metabolism. It converts free fatty acids into acyl-CoA, which serves as a substrate for both β-oxidation and lipid synthesis.^[50]^ Previous studies have shown that the OIP5 gene regulates ACSL1 expression and affects the lipid metabolic phenotype of esophageal squamous cell carcinoma. Activation of ACSL1 is associated with enhanced tumor cell proliferation, migration, and resistance to oxidative stress.^[51]^ In ulcerative colitis models, ACSL1 can work with CPT1A to promote FAO and M2 macrophage polarization through the PPAR-γ signaling pathway.^[52]^ In conclusion, based on its structural characteristics and metabolic properties as a MUFA, we hypothesize that 5-dodecenoic acid may influence energy metabolism and cell fate in EC by acting through fatty acid metabolism pathways involving CPT1A and ACSL1.

However, not all alterations in fatty acid metabolism promote cancer progression. Our research revealed that 3-methyladipate levels (β = -0.2918, *P* = .0039, OR = 0.7469) were negatively correlated with EC risk, suggesting that higher levels of this compound may reduce the risk of EC. 3-Methyladipic acid is a dicarboxylic acid, a product of fatty acid ω-oxidation.^[53]^ In fact, fatty acid ω-oxidation has been proposed as a rescue pathway in mitochondrial and peroxisomal FAO disorders. It aims to reduce the detrimental effects caused by the accumulation of fatty acids in these disorders.^[54]^ Additionally, our study demonstrated a significant negative correlation between 3-hydroxy-2-ethylpropionate levels and the risk of EC (β = -0.2536, *P* = .0016, OR = 0.776). This finding suggested that higher levels of 3-hydroxy-2-ethylpropionate are associated with a reduced risk of developing EC. 3-Hydroxy-2-ethylpropionate is a type of hydroxy fatty acid, typically serving as an intermediate product in the fatty acid metabolic pathway. Although there were no prior studies specifically linking 3-hydroxy-2-ethylpropionate to EC or other forms of cancer, our findings highlighted its potential role as a protective factor. This novel correlation warranted further investigation to elucidate the underlying mechanisms by which 3-hydroxy-2-ethylpropionate may exert its protective effects against EC.

Amino acids are fundamental building blocks of proteins and are crucial for cell growth, repair, and signal transduction.^[55]^ Amino acid metabolism includes transamination, deamination, and synthesis. Tumor cells often showed altered amino acid metabolism to support their rapid proliferation and high metabolic demands.^[56]^ Tryptophan is an essential amino acid necessary for protein synthesis in the human body. Tryptophan metabolism is a key part of amino acid metabolism. Dysregulated tryptophan metabolism promoted the progression of various types of cancer.^[57,58]^ One metabolite of tryptophan metabolism is 8-methoxykynurenate, generated from 8-methoxyindole-2,3-dione through enzymatic reactions. Our study found that 8-methoxykynurenate levels were positively correlated with the risk of EC (β = 0.2026, *P* = .0043, OR = 1.2246). Higher levels of this metabolite may increase the risk of EC. These findings are consistent with previous research highlighting the role of altered tryptophan metabolism in cancer development.

Polyamines, synthesized from amino acids, are essential for mammalian cell growth and proliferation. They are involved in DNA replication, RNA transcription, protein synthesis, and post-translational modification.^[59]^ Nucleotides are essential for energy metabolism, signal transduction, and nucleic acid synthesis. Tumor cells show altered polyamine and nucleotide metabolism to support rapid growth, making these pathways potential targets for cancer diagnosis and treatment.^[59,60]^ Our study showed that the spermidine to MTA ratio was positively correlated with EC risk (β = 0.2278, *P* = .0081, OR = 1.2558). Spermidine, derived from ornithine and methionine, is essential for cellular growth. It stabilizes DNA, modulates ion channels, and promotes autophagy.^[61]^ MTA is formed from decarboxylated S-adenosylmethionine in the biosynthesis of spermidine and spermine. It is cleaved by MTA phosphorylase into adenine and 5’-methylthio-5’-deoxyribose-1-phosphate, which are used for the salvage of ATP and methionine, respectively.^[62]^ Additionally, our study found a positive correlation between the ADP to sulfate ratio and EC risk (β = 0.1759, *P* = .0097, OR = 1.1923). ADP is crucial for cellular energy transfer. It is produced from ATP hydrolysis and converted back to ATP through cellular respiration.^[63]^ The ADP to sulfate ratio reflects the metabolic state of tumor cells. Elevated ADP indicates high energy consumption, typical in proliferating tumor cells. This suggests that higher ADP levels relative to sulfate may increase EC risk by disrupting energy metabolism.

In this study, we identified 8 blood metabolites significantly associated with the risk of EC. The results of the MR study were robust, reducing bias and error, making it particularly valuable for assessing long-term risk factors. Therefore, we believe these 8 blood metabolites may be potential early diagnostic and predictive biomarkers for EC. However, our study had inherent limitations. Firstly, although we attempted to minimize the influence of confounding factors as much as possible, we could not entirely eliminate the potential impact of other confounding variables on the results. Secondly, the primary limitation of this study was that it was conducted solely within a European population, which reduced the risk of population stratification bias but limited the generalizability of the findings. Specifically, the results may not be applicable to other ethnic groups, such as Asian or African populations, due to genetic and environmental differences. Thirdly, the lack of data on specific individuals prevented us from conducting more detailed stratified analyses of the population, leading to inaccuracies in inferred conclusions. Lastly, despite employing multiple analytical approaches for validation, the flexible nature of the outcome assessment criteria in this study inevitably increased the probability of false-positive results.

## 5. Conclusions

In summary, our two-sample MR analysis identified causal relationships between 8 out of 1400 metabolites and EC. These results provide new insights into the potential mechanisms by which metabolites influence esophageal tumorigenesis. Furthermore, our study minimized the influence of reverse causality and other confounding factors. This provides a reliable basis and new perspective for exploring the biological basis of EC. These findings may play a crucial role in the early diagnosis and treatment strategies for EC.

## Acknowledgments

Thanks to all authors for their contributions.

## Author contributions

**Data curation:** Liu Ziyang.

**Formal analysis:** Liu Ziyang.

**Investigation:** Liu Ziyang.

**Methodology:** Liu Ziyang, Jinzhou Guo.

**Software:** Liu Ziyang, Jinzhou Guo.

**Supervision:** Jinzhou Guo.

**Visualization:** Liu Ziyang, Jinzhou Guo, Wenxuan Du.

**Validation:** Jinzhou Guo, Wenxuan Du.

**Writing – original draft:** Liu Ziyang.

## Supplementary Material



## References

[1] Yang J Liu X Cao S Dong X Rao S Cai K. Understanding esophageal cancer: the challenges and opportunities for the next decade. Front Oncol. 2020;10:1727. 33014854 10.3389/fonc.2020.01727 PMC7511760

[2] Sung H Ferlay J Siegel RL. Global cancer statistics 2020: GLOBOCAN estimates of incidence and mortality worldwide for 36 cancers in 185 countries. CA Cancer J Clin. 2021;71:209–49. 33538338 10.3322/caac.21660

[3] National Health Commission Of The People’s Republic Of C. Chinese guidelines for diagnosis and treatment of esophageal carcinoma 2018 (English version). Chin J Cancer Res = Chung-kuo yen cheng yen chiu. 2019;31:223–58. 31156297 10.21147/j.issn.1000-9604.2019.02.01 PMC6513746

[4] Morita FH Bernardo WM Ide E. Narrow band imaging versus lugol chromoendoscopy to diagnose squamous cell carcinoma of the esophagus: a systematic review and meta-analysis. BMC cancer. 2017;17:54. 28086818 10.1186/s12885-016-3011-9 PMC5237308

[5] Li X Xu B Yang H Zhu Z. Gut microbiota, human blood metabolites, and esophageal cancer: a mendelian randomization study. Genes. 2024;15:729. 38927665 10.3390/genes15060729 PMC11203100

[6] Lundberg E Lagergren P Mattsson F Lagergren J. Life expectancy in survivors of esophageal cancer compared with the background population. Ann Surg Oncol. 2022;29:2805–11. 35190948 10.1245/s10434-022-11416-4 PMC8989824

[7] Lee S Cohen DJ. Pharmacotherapy for metastatic esophageal cancer: where do we need to improve? Expert Opin Pharmacother. 2019;20:357–66. 30526127 10.1080/14656566.2018.1551881

[8] Wang Z Gao J Xu C. Targeting metabolism to influence cellular senescence a promising anti-cancer therapeutic strategy. Biomed Pharmacother. 2024;177:116962. 38936195 10.1016/j.biopha.2024.116962

[9] Zhu X Wang K Liu G. Metabolic perturbation and potential markers in patients with esophageal cancer. Gastroenterol Res Pract. 2017;2017:5469597. 28512469 10.1155/2017/5469597 PMC5415862

[10] Balonov I Mattis M Jarmusch S. Metabolomic profiling of upper GI malignancies in blood and tissue: a systematic review and meta-analysis. J Cancer Res Clin Oncol. 2024;150:331. 38951269 10.1007/s00432-024-05857-5 PMC11217139

[11] El-Tanani M Rabbani SA El-Tanani Y Matalka II. Metabolic vulnerabilities in cancer: a new therapeutic strategy. Crit Rev Oncol Hematol. 2024;201:104438. 38977145 10.1016/j.critrevonc.2024.104438

[12] Li Q Lin G Zhang K. Hypoxia exposure induces lactylation of Axin1 protein to promote glycolysis of esophageal carcinoma cells. Biochem Pharmacol. 2024;226:116415. 38972426 10.1016/j.bcp.2024.116415

[13] Birney E. Mendelian randomization. Cold Spring Harbor Perspectives Med. 2022;12:a041302. 10.1101/cshperspect.a041302 PMC9121891 34872952

[14] Chen J Yuan S Fu T. Gastrointestinal consequences of type 2 diabetes mellitus and impaired glycemic homeostasis: a mendelian randomization study. Diabetes Care. 2023;46:828–35. 36800530 10.2337/dc22-1385 PMC10091506

[15] Davies NM Holmes MV Smith GD. Reading Mendelian randomisation studies: a guide, glossary, and checklist for clinicians. BMJ (Clinical research ed.). 2018;362:k601. 10.1136/bmj.k601 PMC6041728 30002074

[16] Li Y Liu H Lo CW. On inverse problems in predator-prey models. J Differ Equ. 2024;397:349–76.

[17] Meng P Xu Z Wang X Yin W Liu H. A novel method for solving the inverse spectral problem with incomplete data. J Comput Appl Math. 2025;463:116525.

[18] Li Y Liu H Lo CW. On inverse problems in multi-population aggregation models. J Differ Equ. 2025;414:94–124.

[19] Sakaue S Kanai M Tanigawa Y; FinnGen. A cross-population atlas of genetic associations for 220 human phenotypes. Nat Genet. 2021;53:1415–24. 34594039 10.1038/s41588-021-00931-x PMC12208603

[20] Chen Y Lu T Pettersson-Kymmer U. Genomic atlas of the plasma metabolome prioritizes metabolites implicated in human diseases. Nat Genet. 2023;55:44–53. 36635386 10.1038/s41588-022-01270-1 PMC7614162

[21] Auton A Brooks LD Durbin RM; 1000 Genomes Project Consortium. A global reference for human genetic variation. Nature. 2015;526:68–74. 26432245 10.1038/nature15393 PMC4750478

[22] Cai J He L Wang H. Genetic liability for prescription opioid use and risk of cardiovascular diseases: a multivariable Mendelian randomization study. Addiction (Abingdon, England). 2022;117:1382–91. 34859517 10.1111/add.15767

[23] Shi X Wang T Teng D Hou S Lin N. A mendelian randomization study investigates the causal relationship between immune cell phenotypes and cerebral aneurysm. Front Genet. 2024;15:1333855. 38313677 10.3389/fgene.2024.1333855 PMC10834707

[24] Burgess S Small DS Thompson SG. A review of instrumental variable estimators for Mendelian randomization. Stat Methods Med Res. 2017;26:2333–55. 26282889 10.1177/0962280215597579 PMC5642006

[25] Guo J Si G Si F. Association of immune cells and the risk of esophageal cancer: a Mendelian randomization study in a East Asian population. Medicine (Baltimore). 2024;103:e38064. 38701252 10.1097/MD.0000000000038064 PMC11062746

[26] Pierce BL Ahsan H Vanderweele TJ. Power and instrument strength requirements for Mendelian randomization studies using multiple genetic variants. Int J Epidemiol. 2011;40:740–52. 20813862 10.1093/ije/dyq151 PMC3147064

[27] Burgess S Thompson SG. Interpreting findings from Mendelian randomization using the MR-Egger method. Eur J Epidemiol. 2017;32:377–89. 28527048 10.1007/s10654-017-0255-x PMC5506233

[28] Verbanck M Chen CY Neale B Do R. Detection of widespread horizontal pleiotropy in causal relationships inferred from Mendelian randomization between complex traits and diseases. Nat Genet. 2018;50:693–8. 29686387 10.1038/s41588-018-0099-7 PMC6083837

[29] Chandel NS. Carbohydrate Metabolism. Cold Spring Harbor Perspect Biol. 2021;13:a040618. 10.1101/cshperspect.a040568 PMC7778149 33397651

[30] Santinon G Pocaterra A Dupont S. Control of YAP/TAZ activity by metabolic and nutrient-sensing pathways. Trends Cell Biol. 2016;26:289–99. 26750334 10.1016/j.tcb.2015.11.004

[31] Barba I Carrillo-Bosch L Seoane J. Targeting the warburg effect in cancer: where do we stand? Int J Mol Sci. 2024;25:3142. 38542116 10.3390/ijms25063142 PMC10970388

[32] Liberti MV Locasale JW. The warburg effect: how does it benefit cancer cells? Trends Biochem Sci. 2016;41:211–8. 26778478 10.1016/j.tibs.2015.12.001 PMC4783224

[33] Ediriweera MK Jayasena S. The role of reprogrammed glucose metabolism in cancer. Metabolites. 2023;13:345. 36984785 10.3390/metabo13030345 PMC10051753

[34] Pan S Ding N Ren J. Maltooligosaccharide-forming amylase: characteristics, preparation, and application. Biotechnol Adv. 2017;35:619–32. 28457999 10.1016/j.biotechadv.2017.04.004

[35] Xu HL Zhou X Chen S. Rare sugar L-sorbose exerts antitumor activity by impairing glucose metabolism. Commun Biol. 2023;6:259. 36906698 10.1038/s42003-023-04638-z PMC10008635

[36] Hoy AJ Nagarajan SR Butler LM. Tumour fatty acid metabolism in the context of therapy resistance and obesity. Nat Rev Cancer. 2021;21:753–66. 34417571 10.1038/s41568-021-00388-4

[37] Chandel NS. Lipid metabolism. Cold Spring Harbor Perspect Biol. 2021;13:a040618. 10.1101/cshperspect.a040576 PMC8411952 34470787

[38] Cheng H Wang M Su J. Lipid metabolism and cancer. Life (Basel, Switzerland). 2022;12:784. 35743814 10.3390/life12060784 PMC9224822

[39] Mohamed AH Said NM. Immunohistochemical expression of fatty acid synthase and vascular endothelial growth factor in primary colorectal cancer: a clinicopathological study. J Gastrointest Cancer. 2019;50:485–92. 29681001 10.1007/s12029-018-0104-5

[40] Jiao R Jiang W Xu K Luo Q Wang L Zhao C. Lipid metabolism analysis in esophageal cancer and associated drug discovery. J Pharm Anal. 2024;14:1–15. 38352954 10.1016/j.jpha.2023.08.019 PMC10859535

[41] Guo Y Pan S Ke Y Pan J Li Y Ma H. Seven fatty acid metabolism-related genes as potential biomarkers for predicting the prognosis and immunotherapy responses in patients with esophageal cancer. Vaccines. 2022;10:1721. 36298586 10.3390/vaccines10101721 PMC9610070

[42] Si T Liu D Li L. Lipid identification of biomarkers in esophageal squamous cell carcinoma by lipidomic analysis. Nutr Cancer. 2024;76:608–18. 38753560 10.1080/01635581.2024.2350097

[43] Cui MY Yi X Cao ZZ Zhu D-X Wu J. Targeting strategies for aberrant lipid metabolism reprogramming and the immune microenvironment in esophageal cancer: a review. J Oncol. 2022;2022:4257359. 36106333 10.1155/2022/4257359 PMC9467784

[44] Onkenhout W Venizelos V van der Poel PF van den Heuvel MP Poorthuis BJ. Identification and quantification of intermediates of unsaturated fatty acid metabolism in plasma of patients with fatty acid oxidation disorders. Clin Chem. 1995;41:1467–74. 7586519

[45] Jiang Q Zhang D Hu N. Environmental chemical-induced cardiometabolic disorders: combined epidemiological and experimental evidence. Environ Sci Technol. 2025;59:3853–68. 39977603 10.1021/acs.est.4c09728

[46] Liu Z Liu W Wang W. CPT1A-mediated fatty acid oxidation confers cancer cell resistance to immune-mediated cytolytic killing. Proc Natl Acad Sci USA. 2023;120:e2302878120. 37722058 10.1073/pnas.2302878120 PMC10523454

[47] Tian T Lu Y Lin J. CPT1A promotes anoikis resistance in esophageal squamous cell carcinoma via redox homeostasis. Redox Biol. 2022;58:102544. 36427397 10.1016/j.redox.2022.102544 PMC9692043

[48] Zhao H Cheng X Yan L. APC/C-regulated CPT1C promotes tumor progression by upregulating the energy supply and accelerating the G1/S transition. Cell Commun Signaling. 2024;22:283. 10.1186/s12964-024-01657-z PMC11112774 38783346

[49] Su W Xu F Zhong J. Screening of CPT1A-targeting lipid metabolism modulators using mitochondrial membrane chromatography. ACS Appli Materials Interfaces. 2024;16:13234–46. 10.1021/acsami.3c18102 38411590

[50] Ma Y Zha J Yang X. Long-chain fatty acyl-CoA synthetase 1 promotes prostate cancer progression by elevation of lipogenesis and fatty acid beta-oxidation. Oncogene. 2021;40:1806–20. 33564069 10.1038/s41388-021-01667-y PMC8842993

[51] Cui MY Yi X Zhu DX Wu J. Identification of differentially expressed genes related to the lipid metabolism of esophageal squamous cell carcinoma by integrated bioinformatics analysis. Curr Oncol (Toronto, Ont.). 2022;30:1–18. 10.3390/curroncol30010001 PMC9858068 36661650

[52] Li J Zou P Xiao R Wang Y. Indole-3-propionic acid alleviates DSS-induced colitis in mice through macrophage glycolipid metabolism. Int Immunopharmacol. 2025;152:114388. 40086057 10.1016/j.intimp.2025.114388

[53] Ranea-Robles P Houten SM. The biochemistry and physiology of long-chain dicarboxylic acid metabolism. Biochem J. 2023;480:607–27. 37140888 10.1042/BCJ20230041 PMC10214252

[54] Wanders RJ Komen J Kemp S. Fatty acid omega-oxidation as a rescue pathway for fatty acid oxidation disorders in humans. FEBS J. 2011;278:182–94. 21156023 10.1111/j.1742-4658.2010.07947.x

[55] Ling ZN Jiang YF Ru JN Lu J-H Ding B Wu J. Amino acid metabolism in health and disease. Signal Transduction Targeted Ther. 2023;8:345. 10.1038/s41392-023-01569-3 PMC10497558 37699892

[56] Bastings J van Eijk MH Olde Damink SW. d-amino acids in health and disease: a focus on cancer. Nutrients. 2019;11:2205. 31547425 10.3390/nu11092205 PMC6770864

[57] Venkateswaran N Lafita-Navarro MC Hao YH. MYC promotes tryptophan uptake and metabolism by the kynurenine pathway in colon cancer. Genes Develop. 2019;33:1236–51. 31416966 10.1101/gad.327056.119 PMC6719621

[58] Liu X Zhang M Liu X. Urine metabolomics for Renal Cell Carcinoma (RCC) prediction: tryptophan metabolism as an important pathway in RCC. Front Oncol. 2019;9:663. 31380290 10.3389/fonc.2019.00663 PMC6653643

[59] Xuan M Gu X Li J Huang D Xue C He Y. Polyamines: their significance for maintaining health and contributing to diseases. Cell Commun Signaling. 2023;21:348. 10.1186/s12964-023-01373-0 PMC10694995 38049863

[60] Holbert CE Cullen MT Casero RA Jr. Stewart TM. Polyamines in cancer: integrating organismal metabolism and antitumour immunity. Nat Rev Cancer. 2022;22:467–80. 35477776 10.1038/s41568-022-00473-2 PMC9339478

[61] Minois N. Molecular basis of the ‘anti-aging’ effect of spermidine and other natural polyamines - a mini-review. Gerontology. 2014;60:319–26. 24481223 10.1159/000356748

[62] Savarese TM Ghoda LY Dexter DL Parks RE. Conversion of 5’-deoxy-5’-methylthioadenosine and 5’-deoxy-5’-methylthioinosine to methionine in cultured human leukemic cells. Cancer Res. 1983;43:4699–702. 6411330

[63] Pelleg A. Extracellular adenosine 5’-triphosphate in pulmonary disorders. Biochem Pharmacol. 2021;187:114319. 33161021 10.1016/j.bcp.2020.114319

