# Supplementary material for: Causal association of metabolic biomarkers and the risk of esophageal cancer: A 2-sample Mendelian randomization study
Source: Medicine (Baltimore). 2025 Aug 1;104(31):e43295. doi: 10.1097/MD.0000000000043295 (PMC12324000; doi:10.1097/MD.0000000000043295)

Supplementary figure 1: Scatter plots of causal relationships between 9 kinds of blood metabolites and EC.


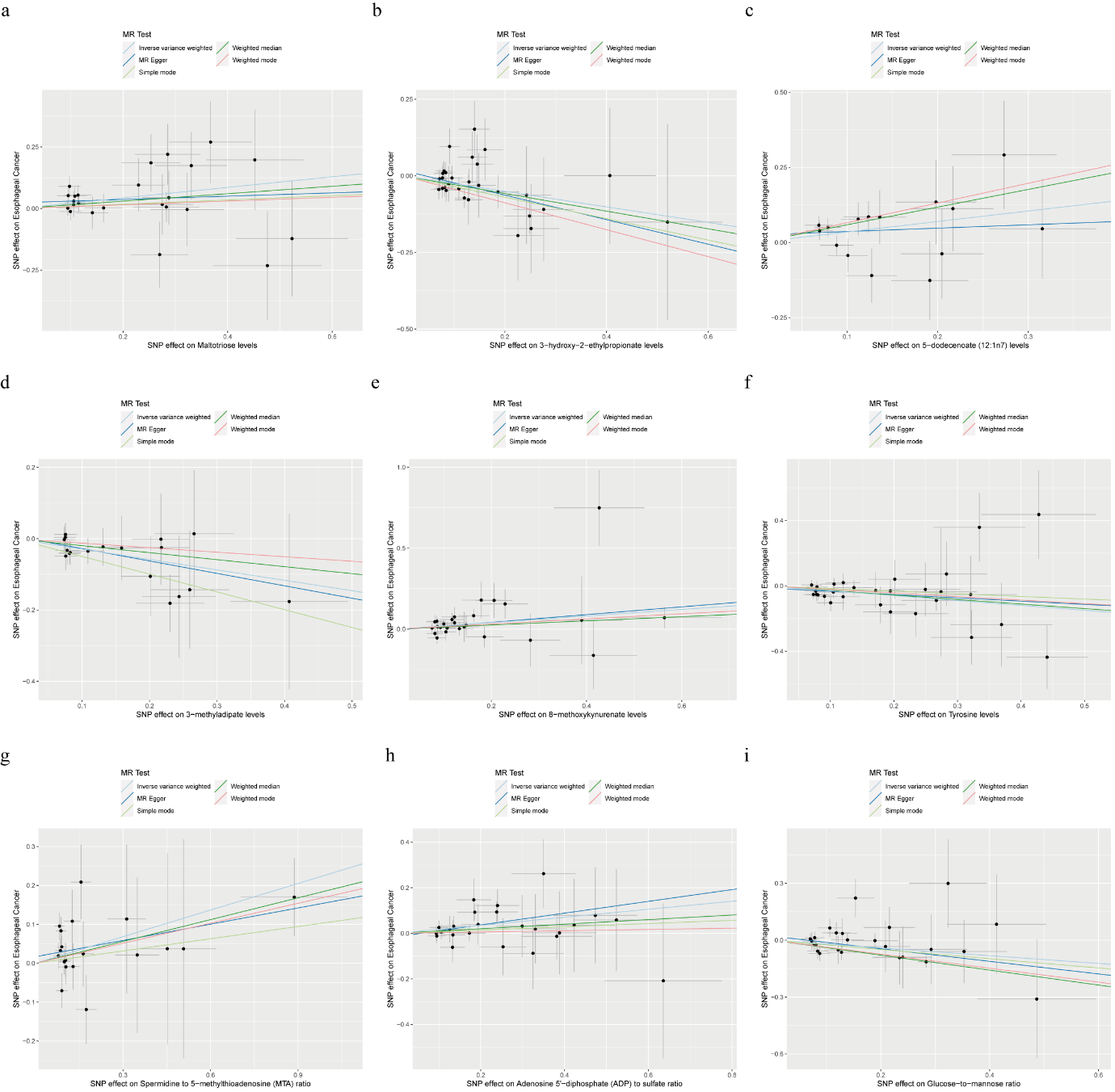


**Supplementary figure 2**: MR leave-one-out sensitivity analysis of causal relationships between 9 kinds of blood metabolites and EC.


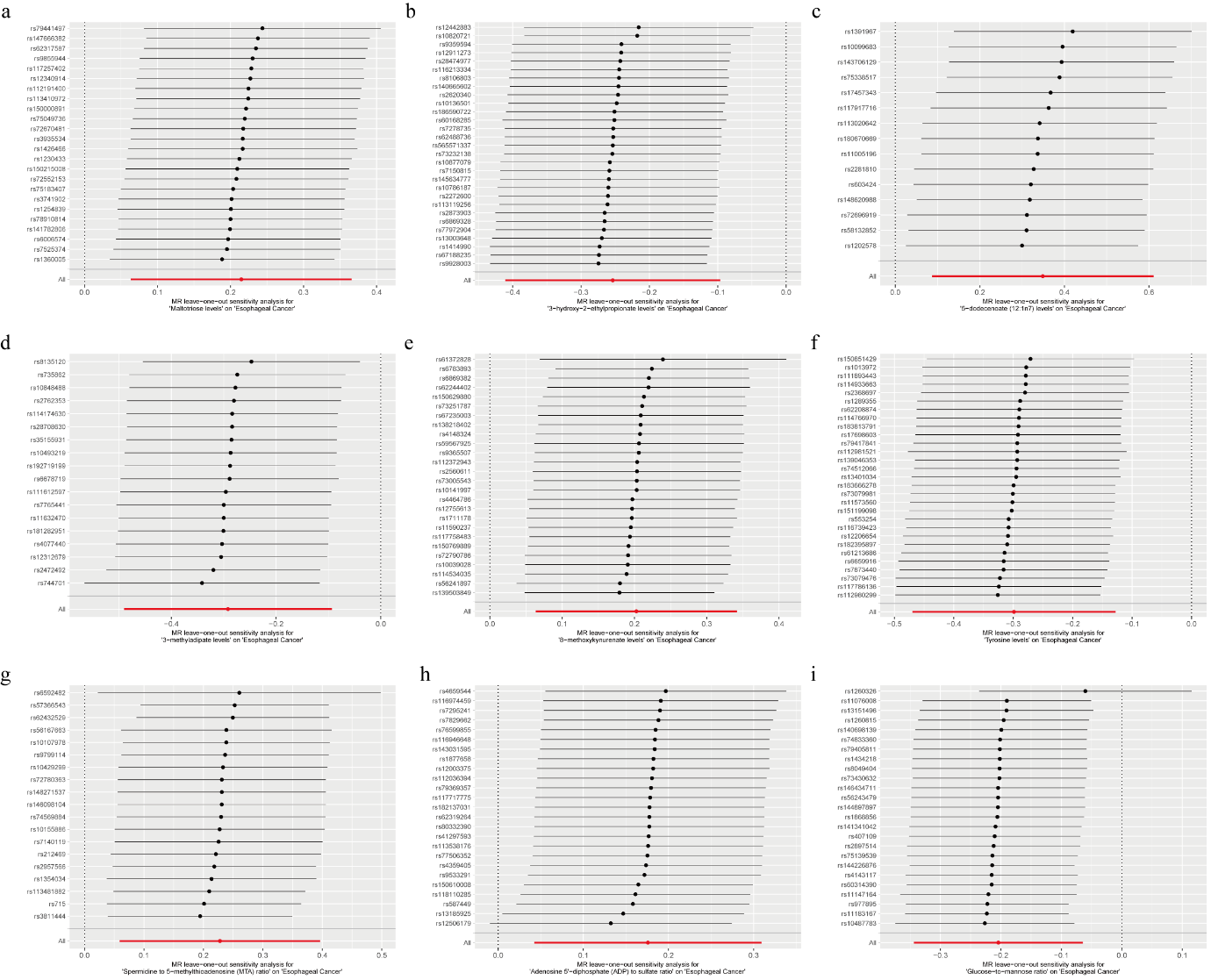


**Supplementary figure 3**: Funnel plot to assess heterogeneity of causal relationships between 9 kinds of blood metabolites and EC. The blue linerepresents the inverse variance weighted estimate, and the dark blue line represents the MR-Egger estimate.


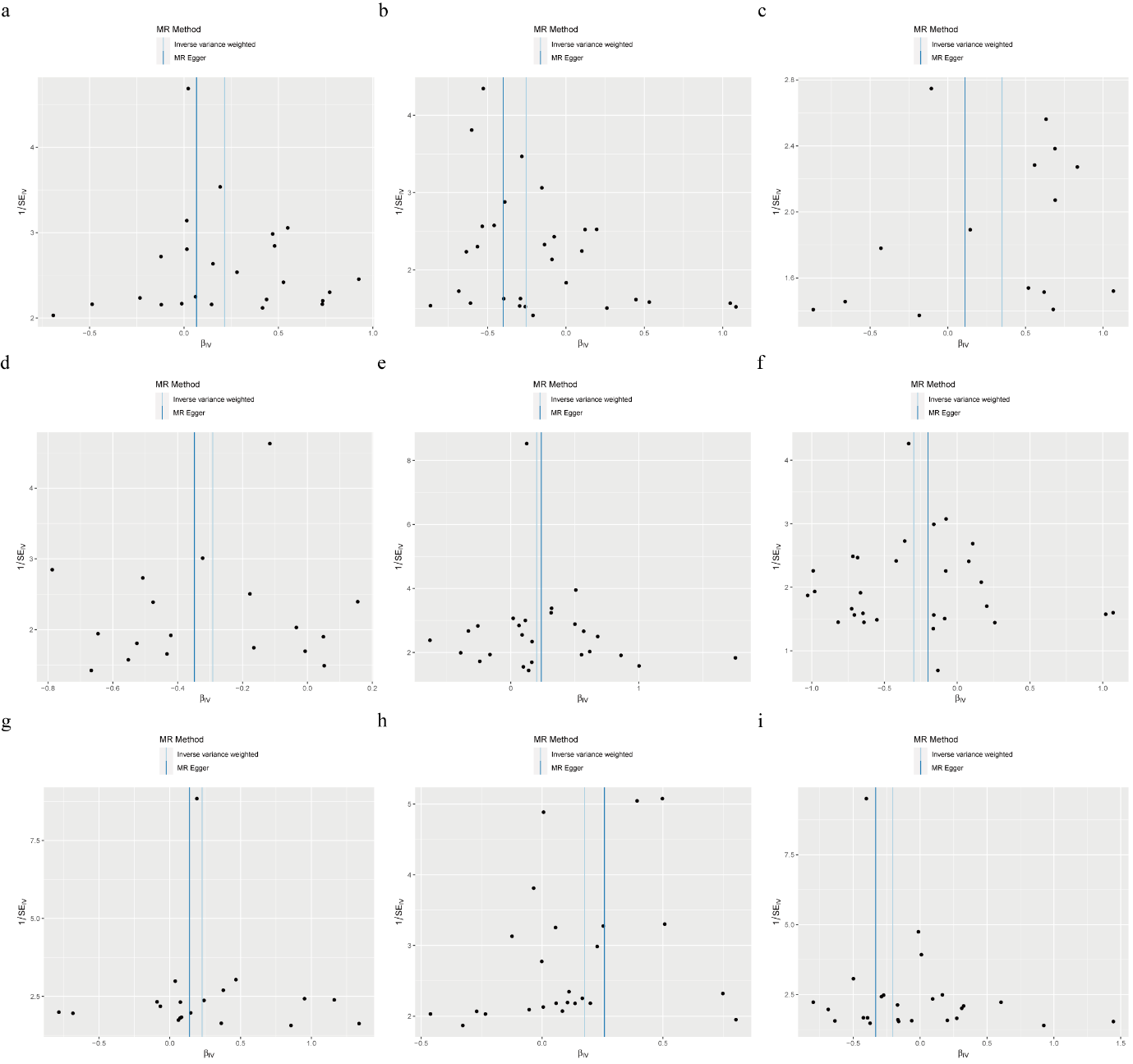


**Supplementary figure 4**: Scatter plots of causal relationships between EC and 9 kinds of blood metabolites.


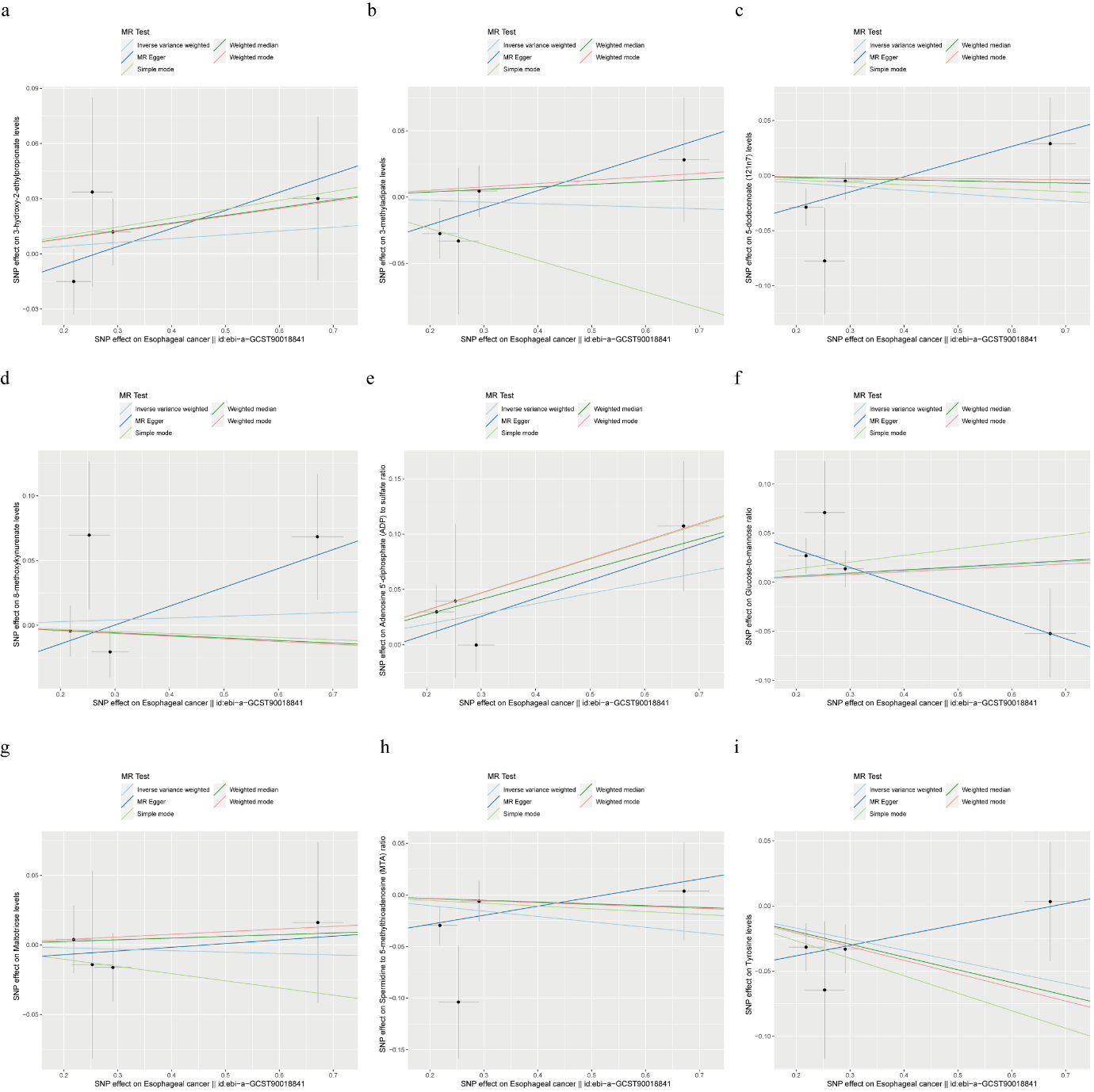


**Supplementary figure 5**: MR leave-one-out analysis of causal relationships between EC and 9 kinds of blood metabolites.


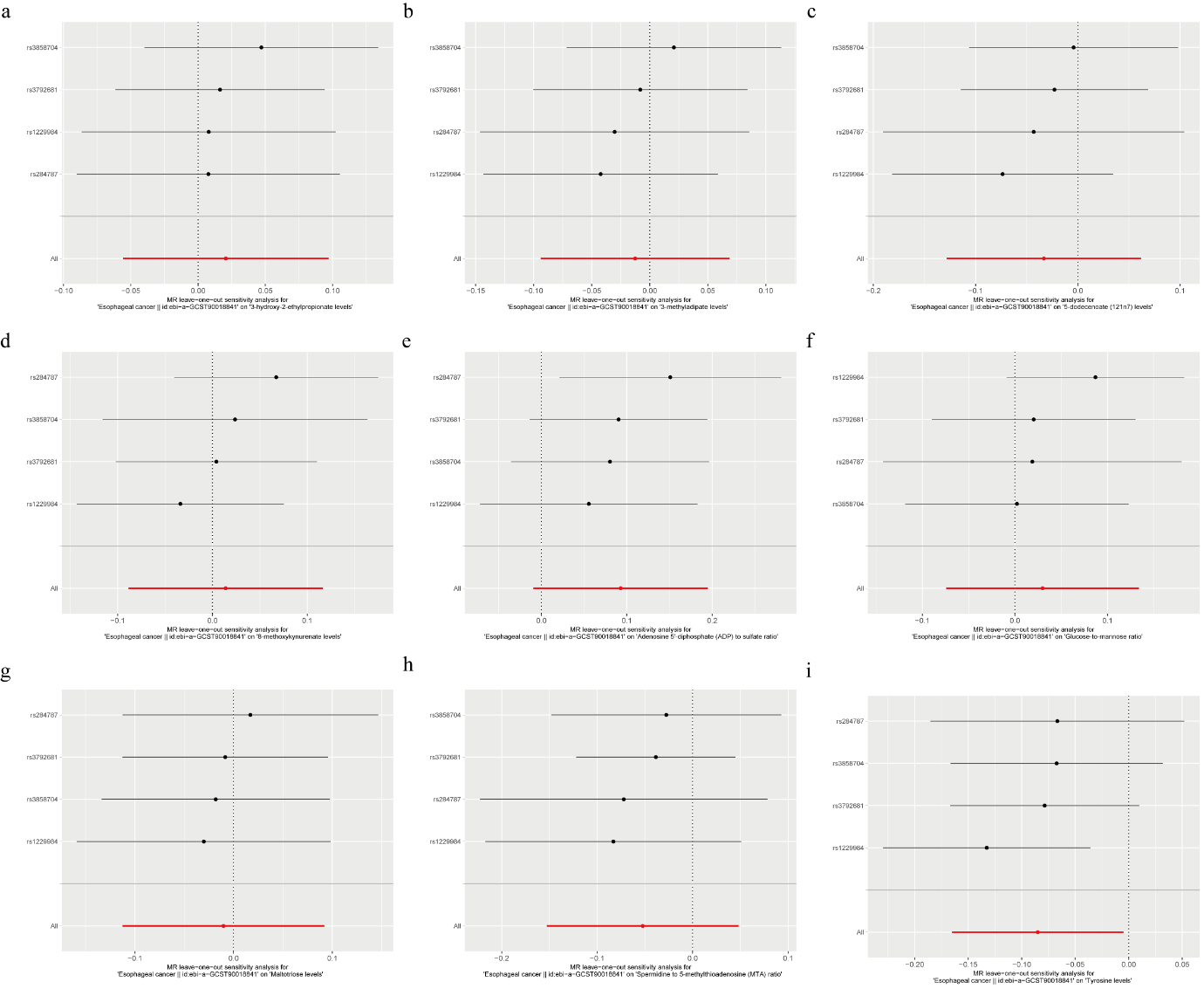


**Supplementary figure 6**: Funnel plot to assess heterogeneity of causal relationships between EC and 9 kinds of blood metabolites. The blue linerepresents the inverse variance weighted estimate, and the dark blue line represents the MR-Egger estimate.


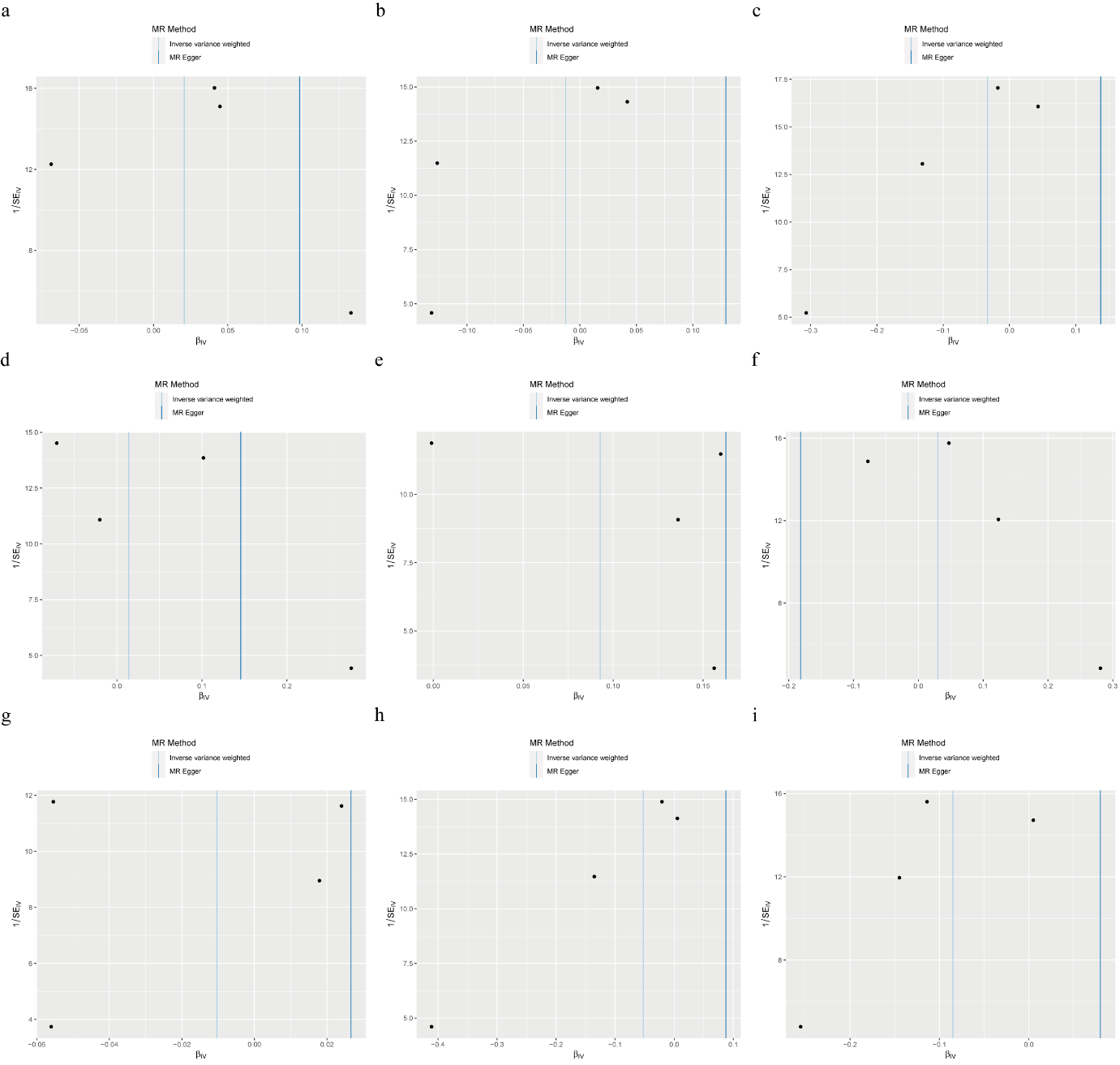

Supplement: Supplementary file 2 [file medi-104-e43295-s002.docx]
